# Supplementary material for: Difference in adsorbable organic halogen formation between phenolic and non-phenolic lignin model compounds in chlorine dioxide bleaching
Source: R Soc Open Sci. 2019 Oct 9;6(10):191202. doi: 10.1098/rsos.191202 (PMC6837227; doi:10.1098/rsos.191202)
Supplement: ESM for Fig. 1, 2, 3, 4, 5, 6 [file rsos191202supp1.docx]

Effect of pH, ClO_2_ concentration, lignin model compound concentration, temperature on AOX formation

ESM for Fig. 1a

| Time (min) | pH | | | | |
| --- | --- | --- | --- | --- | --- |
|  | 2.00 | 2.50 | 3.00 | 3.50 | 4.00 |
| 1 | 387.13 | 371.67 | 331.00 | 303.45 | 281.26 |
| 2 | 416.31 | 373.36 | 343.20 | 309.75 | 282.43 |
| 3 | 417.14 | 384.19 | 354.64 | 320.25 | 293.88 |
| 4 | 426.98 | 388.32 | 370.04 | 325.54 | 305.03 |
| 6 | 435.00 | 407.69 | 373.56 | 333.91 | 316.26 |
| 8 | 446.44 | 419.02 | 377.08 | 339.15 | 319.33 |
| 10 | 451.04 | 421.26 | 385.44 | 348.60 | 320.09 |
| 15 | 459.32 | 425.00 | 386.32 | 362.25 | 328.30 |
| 30 | 467.02 | 434.20 | 396.88 | 375.91 | 349.00 |

ESM for Fig. 1b

| Time (min) | pH | | | | |
| --- | --- | --- | --- | --- | --- |
|  | 2.00 | 2.50 | 3.00 | 3.50 | 4.00 |
| 1 | 338.76 | 292.81 | 287.69 | 278.85 | 249.31 |
| 2 | 529.43 | 464.21 | 429.08 | 399.33 | 361.80 |
| 3 | 609.04 | 563.20 | 532.29 | 497.20 | 460.84 |
| 4 | 640.40 | 585.44 | 543.43 | 502.08 | 473.44 |
| 6 | 664.39 | 590.00 | 550.34 | 518.29 | 490.50 |
| 8 | 691.33 | 632.40 | 585.20 | 546.79 | 495.92 |
| 10 | 694.24 | 640.09 | 605.02 | 579.00 | 500.42 |
| 15 | 711.00 | 648.18 | 610.24 | 590.10 | 529.24 |
| 30 | 718.34 | 668.30 | 624.99 | 602.24 | 536.40 |

ESM for Fig. 2a

| Time (min) | The concentration of ClO_2_ (mmol·L^-1^) | | | | |
| --- | --- | --- | --- | --- | --- |
|  | 24.80 | 49.60 | 73.40 | 99.20 | 124.00 |
| 1 | 60.62 | 160.65 | 194.04 | 278.34 | 331.03 |
| 2 | 63.15 | 166.60 | 209.09 | 299.64 | 343.22 |
| 3 | 63.24 | 167.30 | 213.14 | 303.61 | 354.64 |
| 4 | 63.40 | 170.45 | 224.52 | 308.44 | 370.04 |
| 6 | 64.44 | 170.82 | 226.49 | 312.80 | 373.56 |
| 8 | 69.00 | 176.05 | 231.54 | 314.84 | 377.08 |
| 10 | 71.21 | 180.25 | 233.96 | 320.01 | 385.44 |
| 15 | 72.01 | 182.70 | 235.77 | 321.63 | 386.32 |
| 30 | 72.41 | 186.93 | 247.35 | 324.80 | 396.88 |

ESM for Fig. 2b

| Time (min) | The concentration of ClO_2_ (mmol·L^-1^) | | | | |
| --- | --- | --- | --- | --- | --- |
|  | 24.80 | 49.60 | 73.40 | 99.20 | 124.00 |
| 1 | 76.40 | 125.25 | 153.25 | 227.94 | 288.21 |
| 2 | 80.41 | 164.22 | 285.18 | 386.36 | 429.24 |
| 3 | 107.35 | 184.02 | 331.01 | 418.20 | 532.03 |
| 4 | 114.77 | 206.11 | 345.88 | 441.42 | 543.25 |
| 6 | 122.10 | 228.10 | 373.01 | 467.22 | 550.00 |
| 8 | 137.05 | 241.90 | 387.69 | 483.18 | 584.89 |
| 10 | 138.16 | 248.20 | 394.42 | 489.01 | 604.82 |
| 15 | 140.03 | 260.06 | 400.19 | 490.02 | 610.28 |
| 30 | 164.89 | 297.11 | 407.08 | 515.42 | 625.40 |

ESM for Fig. 3a

| Time (min) | The concentration of VA (mmol·L^-1^) | | | | |
| --- | --- | --- | --- | --- | --- |
|  | 31.00 | 46.50 | 62.00 | 77.50 | 93.00 |
| 1 | 210.09 | 286.90 | 331.10 | 360.43 | 405.61 |
| 2 | 213.42 | 299.75 | 343.20 | 381.21 | 419.89 |
| 3 | 231.53 | 301.15 | 354.64 | 397.24 | 427.65 |
| 4 | 233.45 | 305.91 | 370.04 | 402.02 | 429.65 |
| 6 | 238.99 | 312.55 | 373.56 | 405.63 | 449.80 |
| 8 | 243.19 | 316.35 | 377.08 | 409.21 | 451.12 |
| 10 | 245.00 | 332.53 | 385.44 | 421.20 | 453.73 |
| 15 | 251.20 | 337.25 | 386.32 | 428.43 | 465.40 |
| 30 | 252.29 | 339.15 | 396.88 | 440.43 | 479.73 |

ESM for Fig. 3b

| Time (min) | The concentration of VE (mmol·L^-1^) | | | | |
| --- | --- | --- | --- | --- | --- |
|  | 31.00 | 46.50 | 62.00 | 77.50 | 93.00 |
| 1 | 238.33 | 265.24 | 288.42 | 309.54 | 325.51 |
| 2 | 383.02 | 417.10 | 429.02 | 483.70 | 487.55 |
| 3 | 411.00 | 488.18 | 532.20 | 534.68 | 558.84 |
| 4 | 420.20 | 501.35 | 543.45 | 553.33 | 587.47 |
| 6 | 473.38 | 533.09 | 550.25 | 577.53 | 623.83 |
| 8 | 474.09 | 540.41 | 585.02 | 612.81 | 635.92 |
| 10 | 474.33 | 563.24 | 605.29 | 634.90 | 655.90 |
| 15 | 498.03 | 573.04 | 610.34 | 635.89 | 663.54 |
| 30 | 523.23 | 589.11 | 624.22 | 642.09 | 672.32 |

ESM for Fig. 4a

| Time (min) | Reaction temperature (K) | | | | |
| --- | --- | --- | --- | --- | --- |
|  | 323 | 328 | 333 | 338 | 343 |
| 1 | 207.90 | 234.23 | 281.04 | 316.40 | 331.22 |
| 2 | 212.82 | 243.37 | 306.29 | 334.87 | 343.20 |
| 3 | 212.84 | 252.40 | 312.17 | 347.69 | 354.64 |
| 4 | 222.62 | 255.52 | 314.40 | 353.03 | 370.04 |
| 6 | 223.31 | 261.10 | 317.24 | 362.29 | 373.56 |
| 8 | 234.50 | 267.44 | 321.22 | 365.18 | 377.08 |
| 10 | 237.92 | 271.56 | 332.09 | 366.09 | 385.44 |
| 15 | 250.65 | 291.89 | 338.18 | 377.20 | 386.32 |
| 30 | 268.44 | 319.90 | 342.18 | 391.11 | 396.88 |

ESM for Fig. 4b

| Time (min) | Reaction temperature (K) | | | | |
| --- | --- | --- | --- | --- | --- |
|  | 323 | 323 | 323 | 323 | 323 |
| 1 | 136.43 | 155.00 | 180.41 | 249.20 | 288.07 |
| 2 | 210.20 | 251.41 | 259.63 | 310.24 | 429.48 |
| 3 | 223.22 | 291.87 | 352.40 | 384.44 | 532.22 |
| 4 | 265.58 | 315.69 | 371.80 | 422.06 | 543.29 |
| 6 | 299.09 | 372.91 | 399.33 | 442.41 | 550.00 |
| 8 | 306.20 | 379.44 | 436.74 | 480.78 | 584.79 |
| 10 | 371.55 | 406.32 | 447.74 | 514.84 | 604.98 |
| 15 | 374.40 | 420.05 | 452.12 | 535.22 | 610.07 |
| 30 | 388.24 | 440.39 | 486.22 | 558.35 | 625.30 |

ESM for Fig. 5

|  | 1 | 2 | 3 | 4 | 5 | 6 | 7 | 8 | 9 |
| --- | --- | --- | --- | --- | --- | --- | --- | --- | --- |
| Predictions | 39.16 | 78.17 | 108.41 | 150.72 | 177.28 | 216.82 | 276.78 | 314.52 | 327.70 |
| Observations | 40.60 | 80.25 | 94.29 | 166.61 | 178.20 | 209.90 | 282.45 | 306.7. | 306.35 |
|  | 10 | 11 | 12 | 13 | 14 | 15 | 16 | 17 | 18 |
| Predictions | 354.57 | 382.53 | 409.77 | 412.74 | 452.15 | 471.78 | 491.56 | 511.34 | 531.85 |
| Observations | 343.25 | 373.24 | 419.92 | 416.09 | 473.09 | 438.00 | 491.33 | 518.29 | 528.54 |

ESM for Fig. 6

|  | 1 | 2 | 3 | 4 | 5 | 6 | 7 | 8 | 9 |
| --- | --- | --- | --- | --- | --- | --- | --- | --- | --- |
| Predictions | 12.11 | 24.23 | 47.39 | 72.68 | 96.90 | 141.88 | 218.04 | 283.75 | 304.08 |
| Observations | 16.41 | 30.41 | 51.01 | 65.49 | 87.43 | 123.13 | 228.13 | 279.89 | 294.24 |
|  | 10 | 11 | 12 | 13 | 14 | 15 | 16 | 17 | 18 |
| Predictions | 378.62 | 412.13 | 456.12 | 508.77 | 534.86 | 578.94 | 618.20 | 663.08 | 711.32 |
| Observations | 379.24 | 400.43 | 447.58 | 512.08 | 534.07 | 598.87 | 608.69 | 680.28 | 710.97 |
